# Factors related to the postoperative recurrence of lumbar disc herniation treated by percutaneous transforaminal endoscopy: A meta-analysis

**DOI:** 10.3389/fsurg.2022.1049779

**Published:** 2023-01-19

**Authors:** Honglin Li, Wei Deng, Faqiang Wei, Liangmin Zhang, Fan Chen

**Affiliations:** Department of Spine Surgery, Yuechi County Hospital, Guang’an, China

**Keywords:** recurrent lumbar disc herniation, percutaneous transforaminal endoscopic, influencing factor, surgery, lumbar disc herniation

## Abstract

**Objective:**

To explore factors related to the postoperative recurrence of lumbar disc herniation treated by percutaneous transforaminal endoscopy.

**Methods:**

PubMed, EMBASE, Cochrane Library, CNKI, Wanfang database and VIP database were systematically searched from the time of each library's construction to October 20, 2022. The studies that compared the influencing factors of recurrent lumbar disc herniation were included based on the PICO search structure. The Newcastle–Ottawa Scale was used to evaluate the quality of observational studies. The effects of the patient's age, gender, BMI, smoking, drinking, hypertension, diabetes, course of the disease, Pfirrmann grade, and the surgical segment on recurrent lumbar disc herniation were systematically evaluated using Revman 5.3. The odds ratio (OR) and 95% confidence interval (CI) were calculated.

**Results:**

Thirteen studies involving 3,393 patients (323 patients with recurrent lumbar disc herniation) treated with percutaneous transforaminal endoscopy were included in this study. The results of the systematic evaluation showed that the effects of gender, smoking, drinking, hypertension, type of lumbar disc herniation and the surgical segment on recurrent lumbar disc herniation were not statistically significant. However, age ≥60 years (OR = 2.23; 95% CI: 1.13, 4.41), BMI ≥25 (OR = 2.89; 95% CI: 1.23, 6.80), diabetes (OR = 1.73; 95% CI: 1.18, 2.55), course of disease ≥4 years (OR = 2.93; 95% CI: 1.58, 5.43), Pfirrmann grades 3–4 (OR = 3.10; 95% CI: 2.18, 4.40), incomplete removal of nucleus pulposus (OR = 3.26; 95% CI: 1.69, 6.27) and intraoperative fibre breakage (OR = 3.18; 95% CI: 1.56, 6.50) increased the risk of recurrence after treatment.

**Conclusion:**

The recurrence of lumbar disc herniation after percutaneous transforaminal endoscopic treatment is related to demographic characteristics, disease history and surgical conditions. In the future, more high-quality studies are needed to explore the influencing factors of recurrent lumbar disc herniation.

## Introduction

Lumbar disc herniation is a common disease that occurs due to the rupture of the annulus fibrosus resulting in prominent nucleus pulposus and other contents; the compression of the dural sac and nerve root causes lower back and limb numbness, pain and fatigue, resulting in sciatica (1, 2). Compared to conservative treatment, surgery (including open and minimally invasive surgeries) has a good effect on treating lumbar disc herniation and can relieve pain faster (3, 4). Percutaneous transforaminal endoscopic surgery is an effective, minimally invasive surgical treatment. This method treats lumbar disc herniation through the posterolateral transforaminal approach. It removes the nucleus pulposus through the intervertebral foramen to release and relieve the spinal cord and nerve root compression in the spinal canal (5). Compared with traditional open surgery, percutaneous transforaminal endoscopic discectomy for lumbar disc herniation has the advantages of a short preoperative preparation time, a minimally invasive nature, less intraoperative bleeding, a low risk of thrombosis and a low infection rate (6).

However, although the surgical treatment technology has been significantly improved, postoperative recurrence may still occur, leading to recurrent lumbar disc herniation (7). Epidemiological data show that the incidence of recurrent lumbar disc herniation is between 5% and 15% (8, 9). In addition, secondary surgery is more difficult because of the formation of scarring and epidural fibrosis and the patient's enormous physical and psychological burden. Previous studies have shown that recurrent lumbar disc herniation is associated with various factors (10–12), such as age, gender, smoking and lumbar disc degeneration. However, because these findings are not always consistent, it is impossible to draw reliable conclusions on the risk factors for the recurrence of lumbar disc herniation after percutaneous transforaminal endoscopic treatment. Therefore, this study aims to evaluate the effects of different factors on recurrent lumbar disc herniation after percutaneous transforaminal endoscopic treatment by systematically searching Chinese and English databases to provide a theoretical basis for the prevention, treatment and rehabilitation of recurrent lumbar disc herniation.

## Materials and methods

### Search strategy

Following the PRISMA statement, a systematic literature search of PubMed, Embase, Cochrane Library, Web of Science, CINAHL, CNKI and the Wanfang and VIP databases was performed. The search period was from inception to July 30, 2022. The search terms included “lumbar disc herniation or recurrent lumbar disc herniation”, “percutaneous transforaminal endoscopic discectomy or percutaneous endoscopic lumbar discectomy” and “risk factor”. In addition, we manually retrieved the target literature by reading the relevant systematic reviews and references of the included studies. This meta-analysis did not register online, and the protocol was not prepared.

### Inclusion and exclusion criteria

Inclusion criteria: (1) Chinese and English studies published in peer-reviewed journals; (2) the subjects were diagnosed with lumbar disc herniation based on MRI, and the recurrence of lumbar disc herniation after transforaminal endoscopy was not limited to age, gender and duration of illness; (3) information on risk factors for relapsed populations was reported in the literature; (4) case-control studies and cohort studies. Exclusion criteria: (1) non-population study; (2) conference articles, case reports, systematic reviews and other research types; (3) outcome information was insufficient, and data analysis could not be performed; (4) literature research repeated reports; (5) the researchers could not obtain the complete article research.

### Study selection and data extraction

Two reviewers independently reviewed each article's abstracts and full text according to the inclusion of exclusion criteria. For disagreements between the two reviewers, a third reviewer was recruited for discussion until consensus was achieved. After literature screening, two reviewers independently respectively extracted the following information: documentary information, the demographic characteristics of research objects and the influencing factors of recurrent lumbar disc herniation.

### Assessments of methodological quality

The Newcastle–Ottawa Scale (NOS) was used to evaluate the quality of observational studies. The scale was evaluated from eight aspects: the representativeness of the study population, the comparability between groups, the adequacy of the evaluation of the results, the adequacy of the follow-up time and the integrity of the follow-up. The total possible score was nine points; seven points and above indicated high-quality literature, and five points and below indicated low-quality literature.

### Statistical analysis

Revman 5.3 was used to evaluate the quality of diagnostic tests and draw the risk map of bias. The effect size of the count data was expressed by the odds ratio (OR), and the 95% confidence interval (CI) was used to estimate the interval range of the effect size. The heterogeneity test was used to determine the size of heterogeneity by the test of *I*^2^ or Q. If *I*^2^ < 50% or *p* > 0.1, the included literature was considered homogeneous; if *I*^2^ > 50% or *p *≤ 0.1, the included studies were considered largely heterogeneous, and a subgroup meta-analysis was used to explore the source of heterogeneity further. *P *< 0.05 indicated that the difference was statistically significant.

## Results

### Basic characteristics and literature quality evaluation results of included studies

**Figure 1:**
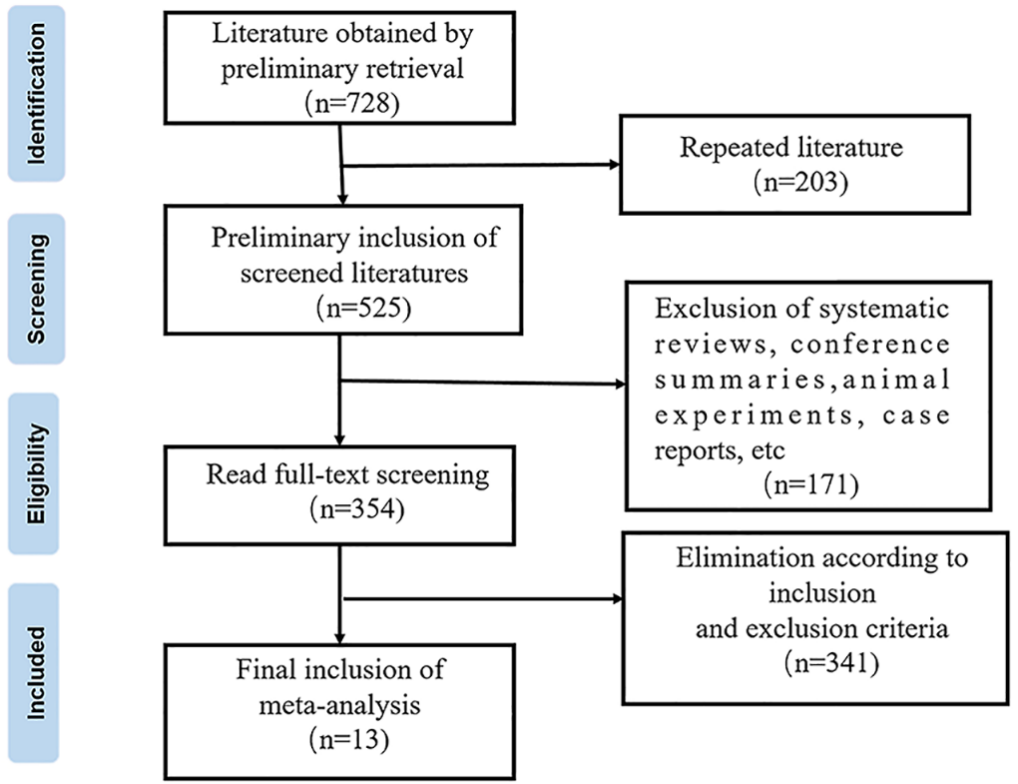
Literature screening flow chart.

After a systematic retrieval and screening of the target literature, 13 studies were included in this study (10–22); the literature screening flow chart is shown in Figure 1. The 13 studies were published from 2007 to 2022, involving 3,393 patients with lumbar disc herniation treated by transforaminal endoscopy, of which 323 were recurrent lumbar disc herniation, and 3,070 patients had no recurrence after treatment. The average age of the patients included in the study was 39.32–63.7 years old, mainly male. The NOS score of the literature quality was 6–9 (mean: 7.5; median: 8.0). Detailed basic characteristics of the included studies and literature quality evaluation results are shown in Table 1.

**Table 1:** Basic characteristics of included studies and literature quality evaluation table.

Study	Study design	Sample size	Relapse	Non-replase	Age (year)	Male (%)	follow up	NOS
Qiao, 2019	Case-control	136	19	117	63.7 ± 5.8	59.6	1 year	7
Zhang, 2021	Case-control	230	29	201	NA	NA	6 months	6
Zhu, 2021	Case-control	120	17	103	48.66 ± 13.57	70	1 year	7
Liang, 2020	Case-control	168	17	151	42.51 ± 14.83	58.9	6 months	8
Fang, 2021	Case-control	324	29	295	NA	55.2	6 months	8
Wei, 2019	Case-control	130	13	117	51.37 ± 6.10	60	1 year	6
Li, 2018	Case-control	378	12	366	52.3 ± 3.6	53.2	18.4 months	8
Chen, 2018	Case-control	100	6	94	39.32 ± 2.13	60	0.25–3.25 year	8
Kim, 2019	Case-control	300	28	272	46.72 ± 15.24	46.3	6–75 months	7
Li, 2022	Case-control	285	19	266	55.96 ± 12.82	56.8	15.5 months	7
Kim, 2007	Case-control	84	42	42	40.9	NA	29.8 months	9
Kong, 2020	Case-control	654	46	608	50.15 ± 12.5	54.3	28.7 months	8
Yu, 2019	Case-control	484	46	438	50.4	52	1–4 years	8

### Effect of age, gender and BMI on recurrent lumbar disc herniation

**Figure 2:**
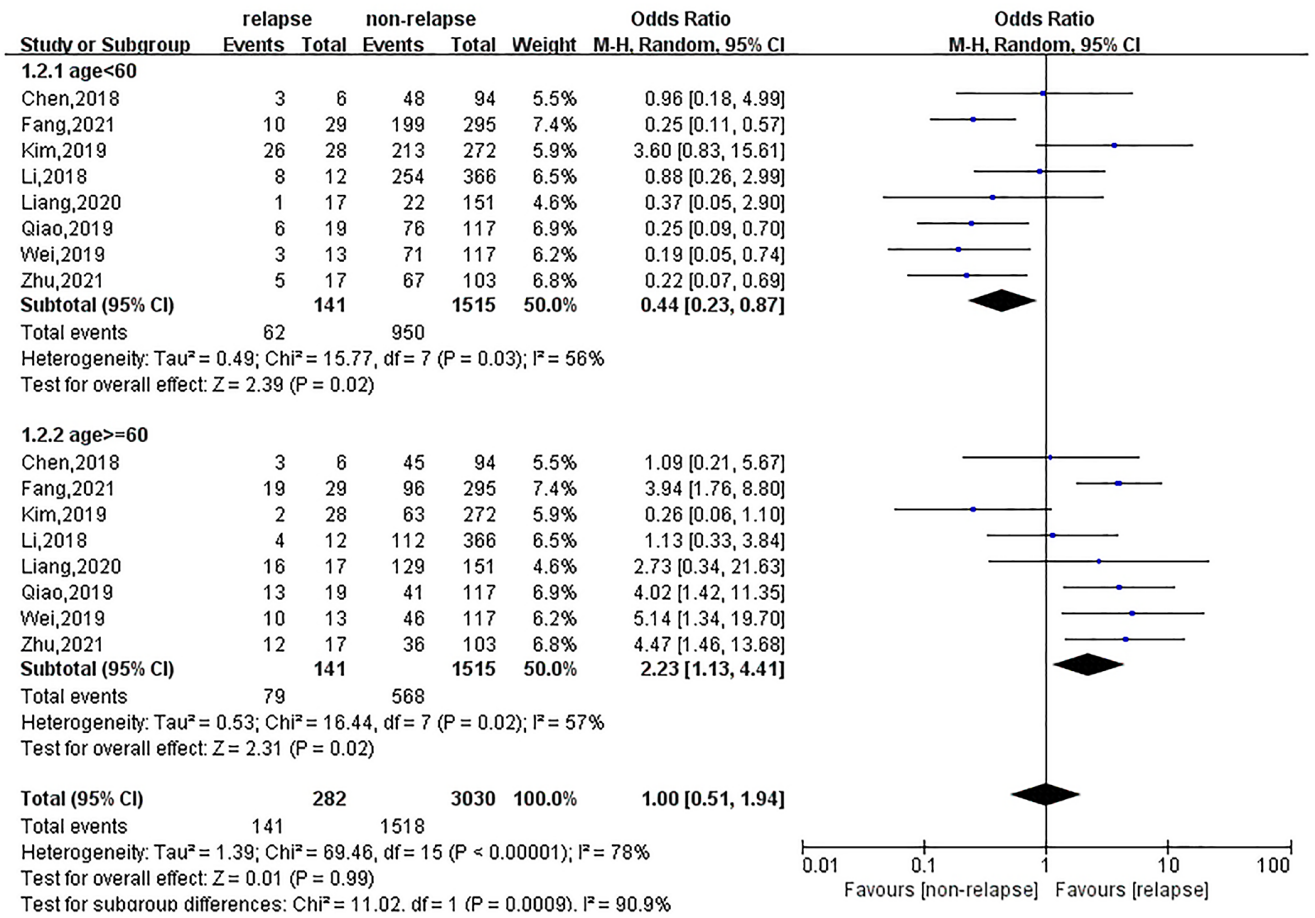
Meta-analysis of the effect of age on recurrent lumbar disc herniation.

**Figure 3:**
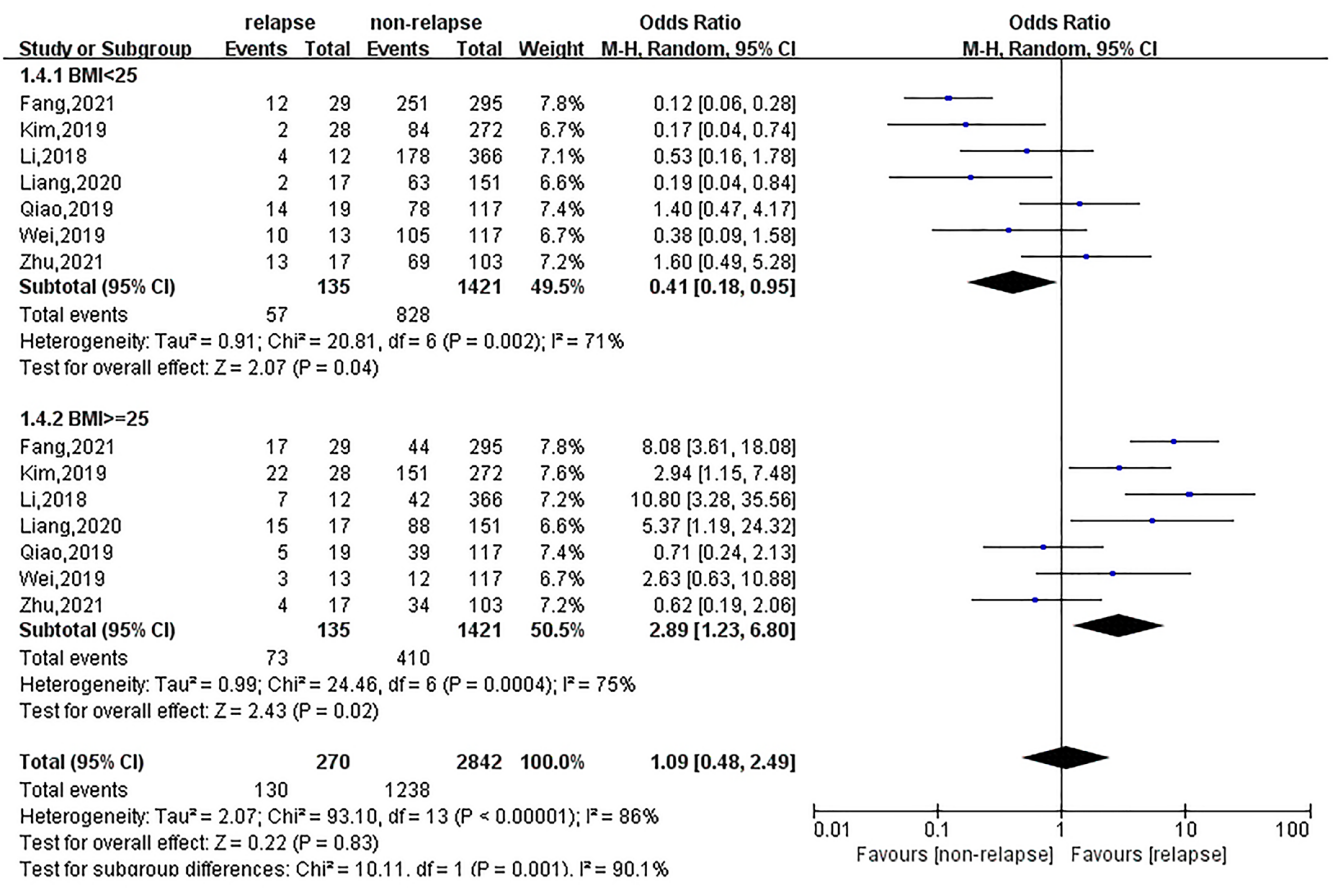
Meta-analysis of the effect of BMI on recurrent lumbar disc herniation.

Eight, ten and seven studies reported the results of age, gender and BMI on the recurrence of lumbar disc herniation after percutaneous transforaminal endoscopic treatment, respectively. Regarding age, 62 patients with recurrence were <60 years old, and 79 patients were ≥60 years old. Patients <60 years old had a lower risk of recurrence after treatment (OR = 0.44; 95% CI: 0.23, 0.87). Age ≥60 years was a risk factor for recurrence; the risk of recurrence after treatment in this population was 2.23-fold (95% CI: 1.13, 4.41), as shown in Figure 2. In terms of gender, there were no significant differences in recurrence between males (OR = 1.01; 95% CI: 0.75, 1.35) and females (OR = 0.97; 95% CI: 0.72, 1.30). Patients with a higher BMI had a higher risk of relapse after treatment, and the risk of recurrent lumbar disc herniation was 2.89 (95% CI: 1.23, 6.80) for BMI ≥25 and 0.41 (95% CI: 0.18, 0.95) for BMI <25, as shown in Figure 3.

### Effects of smoking and drinking on recurrent lumbar disc herniation

The effects of smoking vs. alcohol consumption on recurrent lumbar disc herniation were reported in six and five studies, respectively. According to the heterogeneity evaluation results (*I*^2^ = 54%, *I*^2^ = 0%), the random-effect and fixed-effect models were used to analyse the effect of smoking and drinking, respectively. Meta-analysis showed that there were no statistically significant effects of smoking (OR = 1.41; 95% CI: 0.81, 2.44) and alcohol consumption (OR = 1.43; 95% CI: 0.99, 2.05) on the occurrence of recurrent lumbar disc herniation in patients. However, there was an increased risk of recurrence between smoking and alcohol consumption.

### Effect of hypertension and diabetes mellitus on recurrent lumbar disc herniation

**Figure 4:**
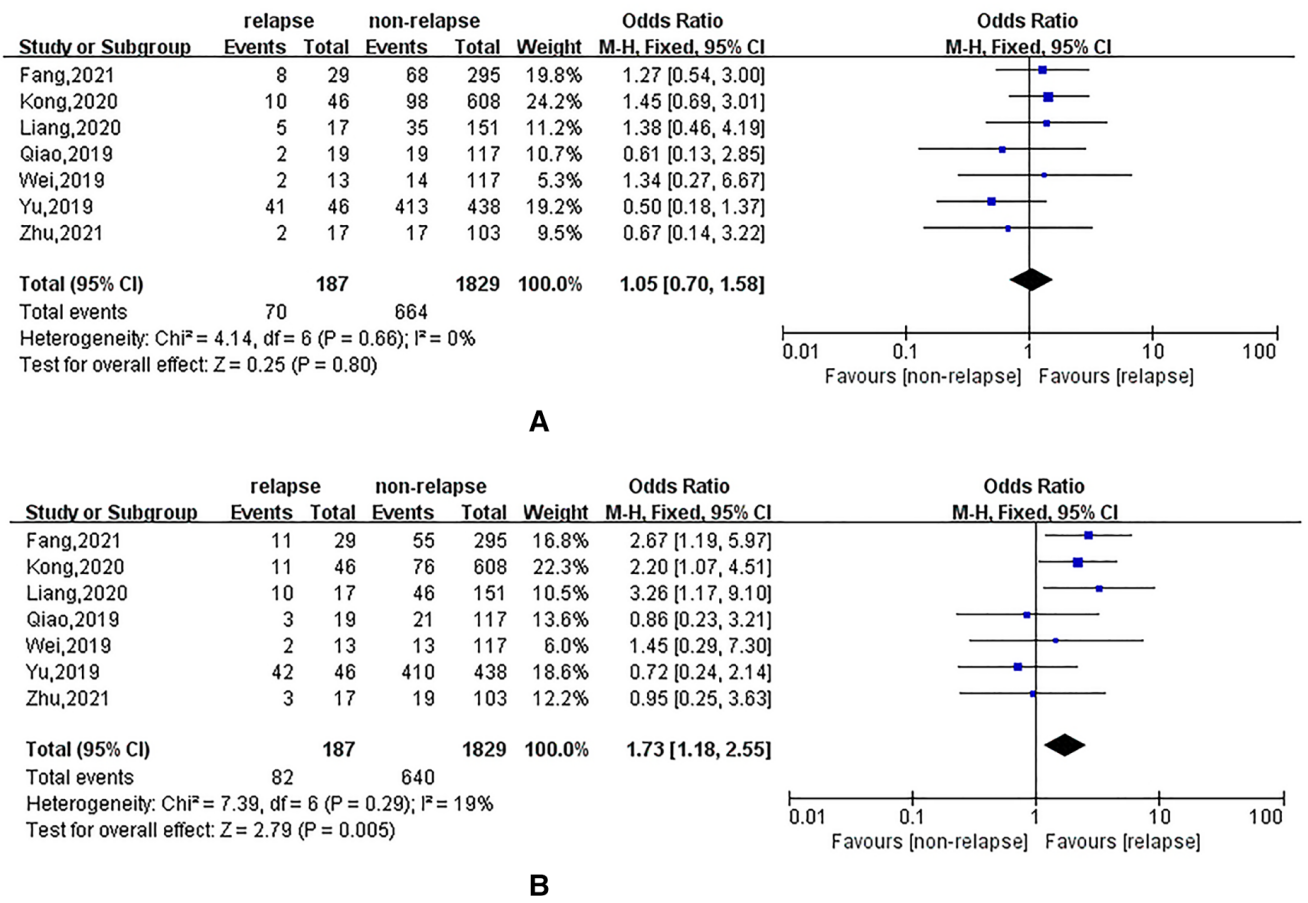
(**A**): meta-analysis of the effect of hypertension on recurrent lumbar disc herniation; (**B**): meta analysis of the effect of diabetes on recurrent lumbar disc.

All seven studies reported the effect of hypertension vs. diabetes on recurrent lumbar disc herniation. Heterogeneity evaluation showed good homogeneity among the included studies (*I*^2^ = 0%, *I*^2^ = 19%), and a fixed-effects model was used for systematic evaluation. A systematic review showed that hypertension (OR = 1.05; 95% CI: 0.70, 1.58; *P* = 0.80) might increase the risk of recurrence in patients, but the difference was not statistically significant, as shown in Figure 4A. Diabetes mellitus significantly increased the risk of recurrent lumbar disc herniation (OR = 1.73; 95% CI: 1.18, 2.55) compared with that of the non-recurrent population, as shown in Figure 4B.

### Effect of course of the disease, type of herniation and pfirrmann grading on recurrent lumbar disc herniation

**Figure 5:**
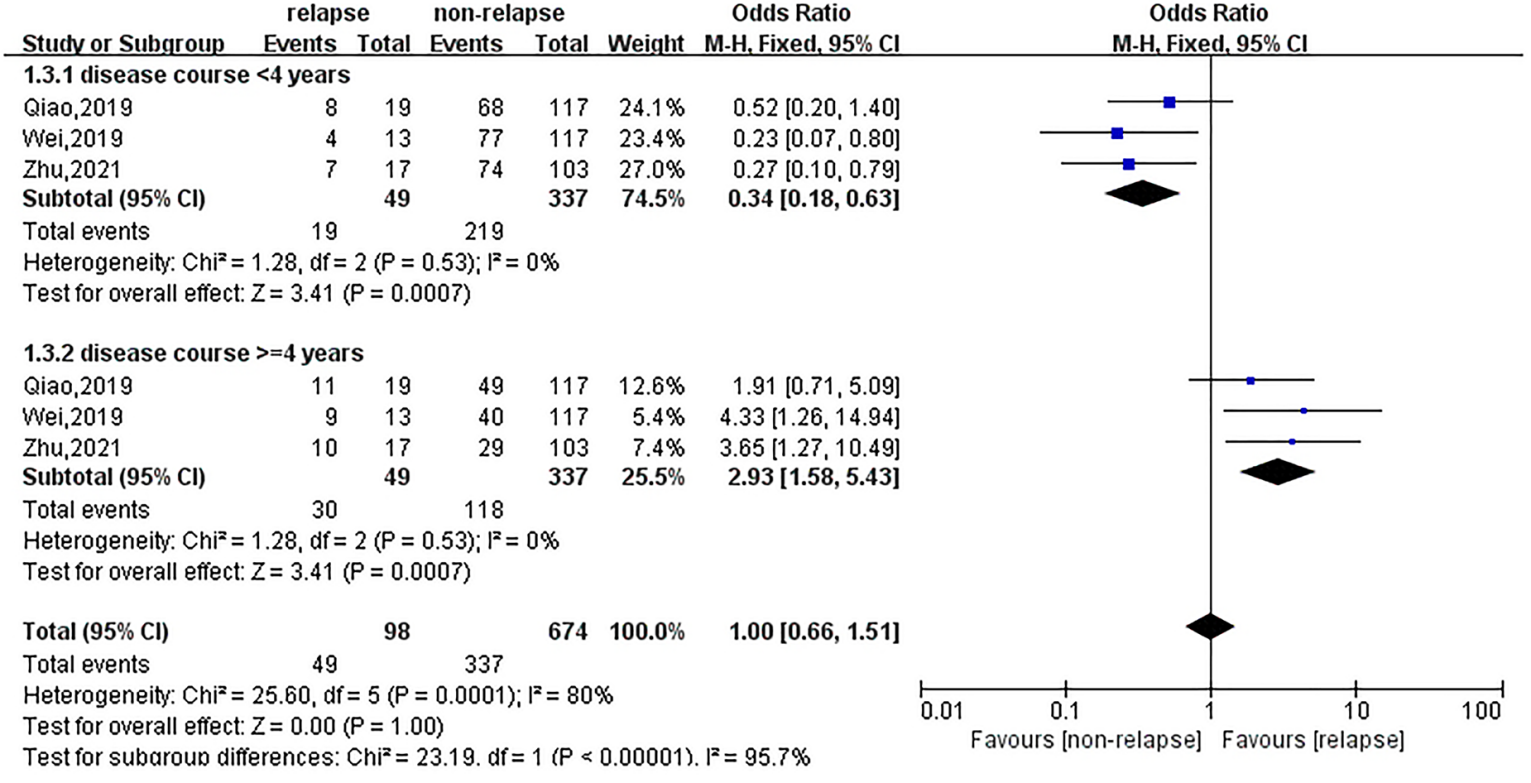
Meta-analysis of the effect of course of disease on recurrent lumbar disc herniation.

**Figure 6:**
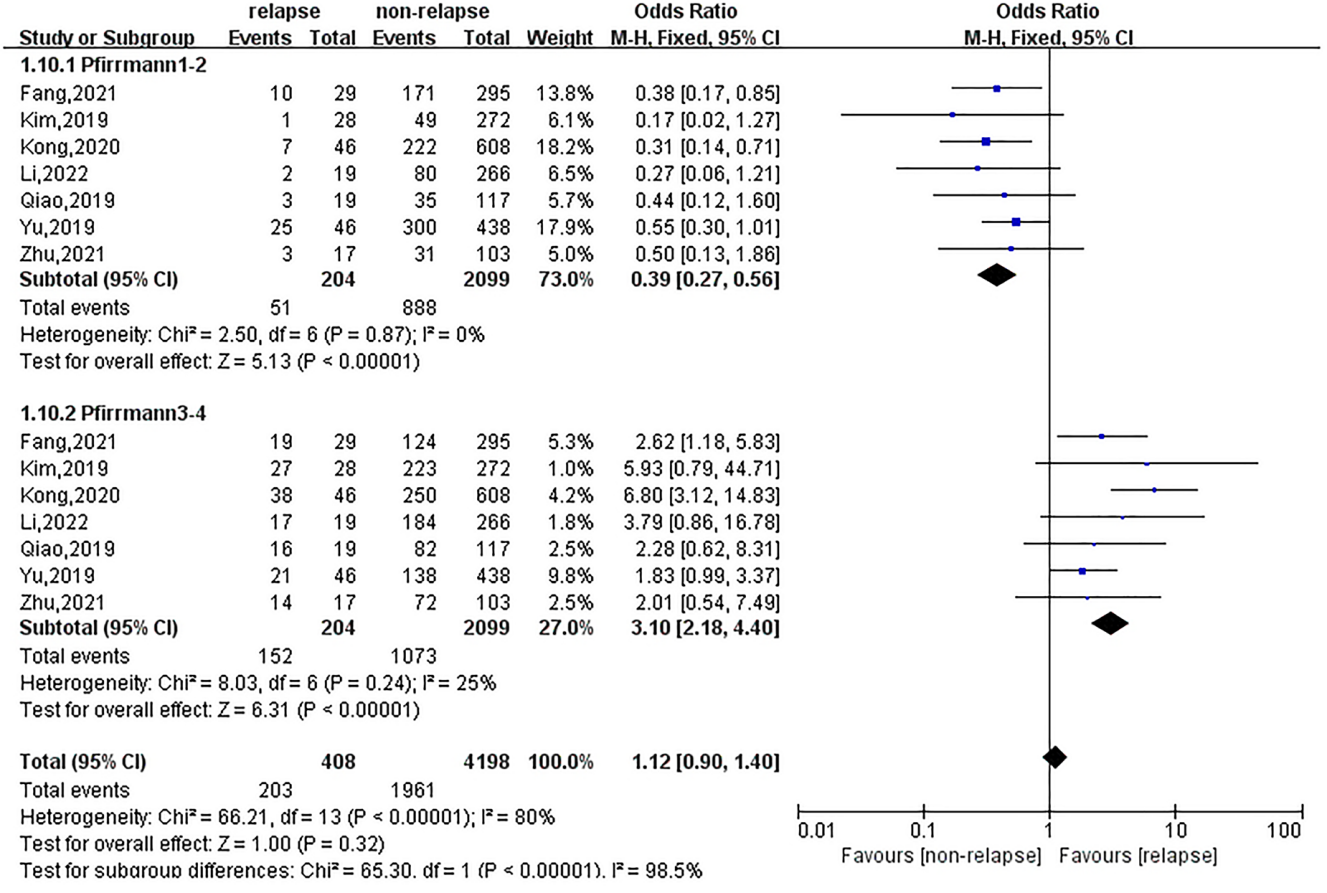
Meta-analysis of the effect of pfirrmann grading on recurrent lumbar disc herniation.

The effects of the disease duration, type of herniation and Pfirrmann grade on recurrent lumbar disc herniation were reported in three, six and seven studies, respectively. Meta-analysis suggested that if the disease duration was ≥4 years, the OR of recurrent lumbar disc herniation after treatment was 2.93 (95% CI: 1.58, 5.43), as shown in Figure 5. There was no statistically significant effect of protrusion, prolapse or mobilisation on recurrence. Patients with Pfirrmann grades 3–4 had a much higher risk of recurrence after treatment than those without recurrence, with an OR of 3.10 (95% CI: 2.18, 4.40), as shown in Figure 6.

### Effect of surgical level, nucleus pulposus removal and fibre damage on recurrent lumbar disc herniation

**Figure 7:**
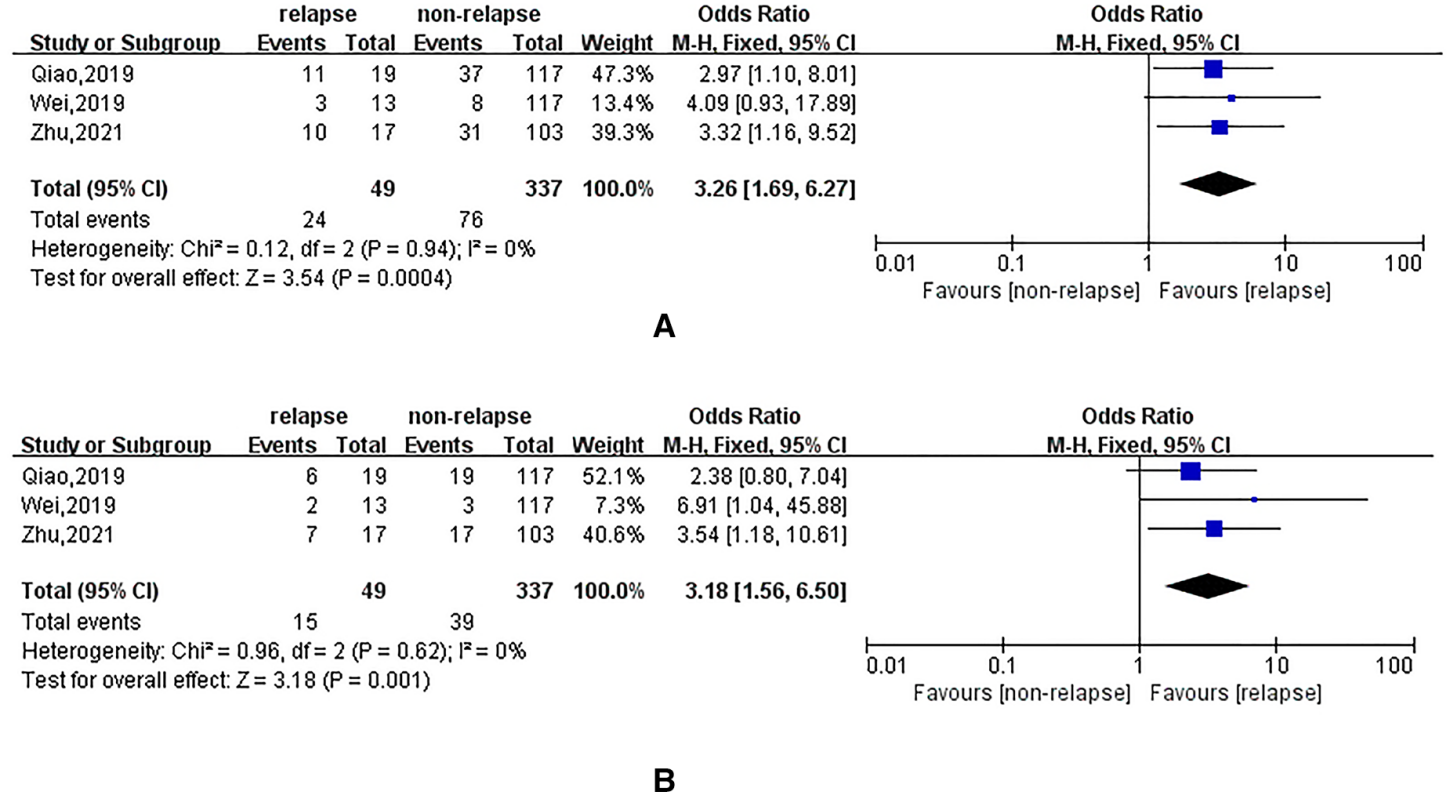
(**A**): meta-analysis of the effect of incomplete nucleus pulposus removal on recurrent lumbar disc herniation; (**B**): meta-analysis of the effect of intraoperative fiber breakage on recurrent lumbar disc herniation.

Eight, three and three studies reported results on the effect of the surgical level of the lumbar disc, incomplete removal of the nucleus pulposus and intraoperative fibrous damage on recurrent lumbar disc herniation in patients, respectively. The heterogeneity evaluation results showed some heterogeneity among the included studies, and the pooled effect size was calculated using a random-effects model. The results of the systematic review showed that there were no statistically significant effects of surgical levels L3–4 (OR = 0.85; 95% CI: 0.34, 2.13), L4–5 (OR = 1.52; 95% CI: 0.76, 3.04) and L5–S1 (OR = 0.72; 95% CI: 0.39, 1.33) on the occurrence of recurrent lumbar disc herniation in patients. In addition, incomplete nucleus pulposus removal (OR = 3.26; 95% CI: 1.69, 6.27) and intraoperative fibrous damage (OR = 3.18; 95% CI: 1.56, 6.50) were associated with an increased risk of post-treatment recurrence, as shown in Figure 7.

## Discussion

After a systematic search and screening, 13 articles were included in this study. The results of the systematic review suggested that gender, smoking, alcohol consumption, hypertension, type of lumbar disc herniation and surgical level had no statistically significant effect on recurrent lumbar disc herniation. However, age ≥60, BMI ≥25, diabetes, duration ≥4 years, Pfirrmann grades 3–4, incomplete nucleus pulposus removal and intraoperative fibrous damage, all of which significantly increase the risk of recurrent lumbar disc herniation, may be risk factors for recurrence after transforaminal endoscopic treatment in patients.

With the great changes brought about by the ageing of the population and social progress in people's lives, lumbar disc herniation has gradually become a common chronic disease in humans, with an incidence of between 2% and 3% (23). Percutaneous transforaminal endoscopic surgery has obvious minimally invasive advantages in treating lumbar disc herniation, but the problem of postoperative recurrence still cannot be ignored (24). Previous studies have shown that older patients are more likely to present with lumbar disc degeneration (25), so age is an important factor in postoperative recurrence. Cinotti et al. (26) suggested that patients with a higher BMI may experience increased disc load due to their lower height, which leads to postoperative recurrence. Zheng et al. (27) found that diabetes may cause decreased nutrient supply and metabolite exchange disorders in the intervertebral disc and accelerate intervertebral disc degeneration in animals. Still, the mechanism of action between diabetes and recurrent lumbar disc herniation remains unclear. In addition, Robinson et al. (28) suggested that diabetes may contribute to increased susceptibility to disc prolapse by comparing discs in diabetic vs. nondiabetic patients. Several studies have found that incomplete nucleus pulposus removal and the integrity of the annulus fibrosus are associated with postoperative recurrence (20, 29, 30), which is heterogeneous with this study. It is important to note that the surgeon's surgical technique is a key factor, foraminoscopy is well-known for its steep learning curve (31), and studies have shown that surgeries performed by surgeons with better surgical techniques and more experience have lower postoperative recurrence rates in patients (30).

There are some limitations to this study that need to be considered. First, this study investigated the effect of multiple factors on recurrent lumbar disc herniation results, and some analyses included fewer studies, which may cause potential bias in the results. Second, because the studies included in this study were case-control studies, causal inference ability was weak. In addition, due to the lack of adequate target literature, we could not conduct a comprehensive systematic evaluation of other influencing factors, like patients' jobs, exercise habits, daily living activities and postoperative education.

## Conclusion

In summary, recurrent lumbar disc herniation after transforaminal endoscopic treatment is associated with multiple influencing factors, such as demographic characteristics, complications, disease history and surgical conditions. Older age, a higher BMI, diabetes mellitus, a higher grade of lumbar disc degeneration, an incomplete nucleus pulposus removal, and intraoperative fibrous damage may be risk factors for recurrent lumbar disc herniation. However, because this study has some limitations, many multicentre and high-quality prospective studies must be carried out to explore the impact of multiple factors and provide a more reasonable theoretical basis for the postoperative management and treatment of patients with lumbar disc herniation.

## Data Availability

The original contributions presented in the study are included in the article/Supplementary Material, further inquiries can be directed to the corresponding author/s.
